# Evaluating the cost effectiveness of donepezil in the treatment of Alzheimer's disease in Germany using discrete event simulation

**DOI:** 10.1186/1471-2377-12-2

**Published:** 2012-02-08

**Authors:** Susanne Hartz, Denis Getsios, Sunning Tao, Steve Blume, Grant Maclaine

**Affiliations:** 1 B214 Baquba Building, Conington Road, SE13 7FF London, UK; 2 United BioSource Corporation, 430 Bedford Street, Suite 300, Lexington Office Park, Lexington, MA 02420, USA; 3 United BioSource Corporation, 185 Dorval Avenue Suite 500, Dorval, Quebec H9S 5J9, Canada; 4 United BioSource Corporation, 7101 Wisconsin Avenue, Bethesda, MD 20814, USA; 5 Becton, Dickinson UK Limited, The Danby Building, Edmund Halley Road, Oxford Science Park, Oxford OX4 4DQ, UK

## Abstract

**Background:**

Previous cost-effectiveness studies of cholinesterase inhibitors have modeled Alzheimer's disease (AD) progression and treatment effects through single or global severity measures, or progression to "Full Time Care". This analysis evaluates the cost-effectiveness of donepezil versus memantine or no treatment in Germany by considering correlated changes in cognition, behavior and function.

**Methods:**

Rates of change were modeled using trial and registry-based patient level data. A discrete event simulation projected outcomes for three identical patient groups: donepezil 10 mg, memantine 20 mg and no therapy. Patient mix, mortality and costs were developed using Germany-specific sources.

**Results:**

Treatment of patients with mild to moderately severe AD with donepezil compared to no treatment was associated with 0.13 QALYs gained per patient, and 0.01 QALYs gained per caregiver and resulted in average savings of €7,007 and €9,893 per patient from the healthcare system and societal perspectives, respectively. In patients with moderate to moderately-severe AD, donepezil compared to memantine resulted in QALY gains averaging 0.01 per patient, and savings averaging €1,960 and €2,825 from the healthcare system and societal perspective, respectively.

In probabilistic sensitivity analyses, donepezil dominated no treatment in most replications and memantine in over 70% of the replications. Donepezil leads to savings in 95% of replications versus memantine.

**Conclusions:**

Donepezil is highly cost-effective in patients with AD in Germany, leading to improvements in health outcomes and substantial savings compared to no treatment. This holds across a variety of sensitivity analyses.

## Background

Alzheimer's disease (AD) is an incurable neurodegenerative disease characterized by cognitive decline, impairment of daily activities and neuropsychiatric symptoms. AD patients lose the ability to perform higher-level daily activities and decrease to being no longer able to perform basic daily necessities such as eating or grooming [1]. Mood swings, apathy, psychosis or agitation are behavioral symptoms commonly observed with AD patients. With increasing severity of the disease dealing with the patients' symptoms can become an increasing burden to caregivers.

A recent study reported the prevalence of dementia in Germany a under 1% of 60-64 year olds and significantly increasing to roughly 20% for those over the 85-89 year olds, up to three quarters of whom had AD [2]. Recent estimates for Germany placed the number of individuals with moderate of severe dementia at just over 1 million, with a projected increase of AD patients in Germany of over 2 million by 2050 [2]. Against the background of an aging population, the German Federal Government has recently intensified its focus on dementia. In 2008, the "Leuchtturmprojekt Demenz" with a budget of 13 million Euro was initiated to improve the evidence-based medical and care service provision for dementia patients [3].

Despite the fact that the benefit of cholinesterase inhibitors have been established by numerous studies [4,5] and that they are a recommended treatment for AD [6], physicians may hesitate to treat patients accordingly given drug acquisition cost considerations [7]. Research on the cost effectiveness of cholinesterase inhibitors is therefore important to provide decision makers with the best possible economic evidence to determine whether concerns over drug acquisition costs are legitimate.

Over the last decade, numerous studies have measured the cost-effectiveness of treatments for AD [8,9], most evaluating the cost-effectiveness of cholinesterase inhibitors. Eight studies have investigated donepezil [10-17], with all but one indicating that donepezil was cost-effective. In Germany, a recent study showed that donepezil was also cost-effective in the German setting, with a base case estimated cost-effectiveness ratio of €4,264 per CDR--Clinical Dementia Rating Scale gained [18].

Previous cost-effectiveness studies have modeled AD progression in terms of cognitive function alone, functional status alone, a single global severity measure, or progression to the need for "Full Time Care". Our study uses an alternative modeling approach to estimate disease progression in terms of correlated changes in cognition, behavior and function. The model was initially constructed for analyses set in the UK [19].

## Methods

The discrete event simulation developed for the evaluation of donepezil's cost-effectiveness in the UK [19,20] was adapted for Germany. The model calculates outcomes from the perspective of both the statutory health insurance and care insurance (Gesetzliche Krankenversicherung/Soziale Pflegeversicherung, GKV/SPV), and from the societal perspective. The GKV/SPV perspective encompasses direct medical costs borne by statutory healthcare insurance including drug costs, costs for monitoring and service provision as well as patient care costs borne by long-term care insurance. The societal perspective comprises both direct and indirect costs, the latter including costs of caregiver time. A discount rate of 3.0% was used for both costs and benefits [21]. In the base case analyses, the time horizon is 10 years in order to capture all potential benefits over the course of the disease.

### Model overview

To allow for individual level modelling, discrete event simulation was used as the modeling technique, capturing heterogeneity in disease progression and other outcomes, as well as tracking correlated changes on multiple domains on continuous rather than aggregated discrete scales. The approach also allows for persistence with treatment to be captured, factoring in time-dependence and the impact of treatment discontinuation on both costs and disease progression in a realistic manner [22-24].

Previous economic evaluations in AD have measured outcomes using highly aggregated health states, and thus were not able to capture the benefits associated with treatment in adequate detail. Furthermore, they have often modeled the disease either based on single domains (e.g., MMSE--Mini-Mental State Examination) or global domains (e.g., CDR--Clinical Dementia Rating Scale), losing the ability to capture the effects of treatment on the full spectrum of AD symptoms. This was driven in part by limitations in data accessible to analysts and the difficulty of tracking progression on multiple measures using traditional Markov model techniques. In addition, most of these models were designed as cohort models with no ability to account for individual characteristics in predicting outcomes, variability in outcomes over the course of the disease or other relevant factors that might influence important determinants of long term outcomes, such as persistence with treatment. The shortcomings of modeling studies in AD have been extensively debated in the literature [8,9,25,26].

Our study adopts an alternative approach in an attempt to overcome some of these limitations. First, it addresses limitations of existing models that focus on a single measure of disease severity alone to model the evolution of AD, by modeling the disease using measures of cognition, behaviour and function. Second, it is an individual simulation that is not encumbered by the limitations imposed by Markovian structures such as an inability to account for individual characteristics by relying on cohort mean values or the use of aggregate health states (e.g., mild, moderate, severe; full-time care, pre-full time care) instead of continuous measurement of disease progression. Finally, as the model employed in this study is an individual patient simulation, it allows for consideration of variation in patient characteristics and disease progression, allows for simulation of persistence with treatment, implementation of clinical stopping rules, and time varying treatment effects and is therefore able to capture disease progression and treatment effects with greater accuracy. The model has been built using ARENA (Version 11) software.

**Figure 1:**
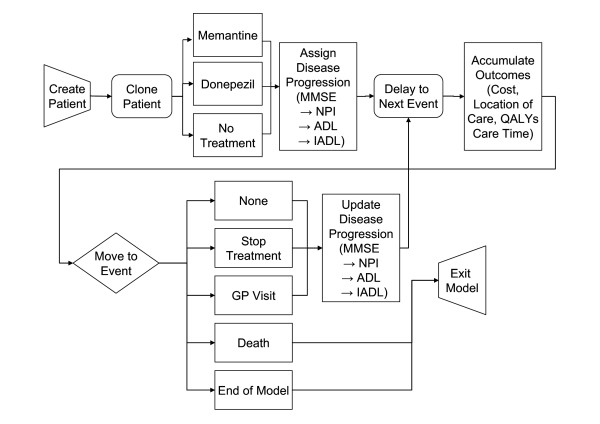
**Simplified representation of the Alzheimer's disease simulation flow**.

Figure 1 provides an overview of the model flow. First, simulated patients are created and individual, unique attributes are assigned. Each patient is then copied twice and the three identical patients are assigned to either no treatment, donepezil 10 mg or memantine 20 mg. Patients are then followed over the course of the simulation with their characteristics updated over time. The simulation measures disease severity based on cognition (using the MMSE), behaviour (using the Neuropsychiatric Inventory, NPI), activities of daily living (ADLs) and instrumental activities of daily living (IADL). In order to preserve the correlation amongst these measures of severity, integrated equations were developed which sequentially predict changes in MMSE, followed by NPI, ADLs, and IADLs. Changes in cognition are first predicted for a simulated individual, which then influence changes in NPI, ADLs, and IADLs. Furthermore, changes in ADL scores are used as part of the prediction of changes in IADL scores. Based on a given patient's characteristics at any point in time, including treatment status and current disease severity, costs, health utilities and caregiver outcomes are calculated and accumulated over the appropriate time period. The model also reports time spent with non-severe symptoms as defined by MMSE, NPI, ADL or IADL scores. MMSE scores below 10 were assumed to represent severe disease according to currently accepted definitions for severe cognitive impairment. For NPI, a cluster analysis of psychiatric symptoms using the NPI of 122 Alzheimer's disease patients in the US [27] was used as the basis for assigning a threshold of 28 for the NPI as representing highly symptomatic behavioral disturbances. IADL and ADL thresholds were arbitrarily set to their mid-point values of 50.

In the simulation, patients can discontinue treatment either based on pre-defined stopping rules, or for other unrelated reasons. As there are currently no defined stopping rules in Germany (e.g., stopping treatment when MMSE scores fall below 10), this option has only been explored in the sensitivity analyses. Mortality is also modeled, although given that neither cholinesterase inhibitors nor memantine have been associated with improvements in survival, time of death is assigned to each individual prior to treatment assignment, thereby ensuring that survival is identical in all groups.

### Data sources

#### Population

An individual patient data set constructed using baseline information from donepezil clinical trials [28-30] is sampled from, to create simulated patients. Characteristics carried in the model include patient age, sex, use of psychiatric medications, MMSE, NPI, ADL- and IADL scores, as well as caregiver age and sex. The trials chosen to provide the sample patients were those that had data on as many target variables as possible, and taken together include all AD severity levels [19].

The age and sex distributions of AD patients in Germany [31] were used to assign sampling weights to individuals in the data set in order to ensure that the age and sex profile of the simulated population was consistent with that of the German AD patient population. Furthermore, analyses are specified according to subgroups of interest, so that sampling is restricted to the relevant population (e.g., patients with mild to moderate AD, as defined by baseline MMSE scores).

#### Disease progression and treatment effects

To improve on existing economic evaluations by including the effects of disease on behavior and function, data were analyzed from the CERAD (Consortium to Establish A Registry for Alzheimer's Disease) registry [32], and seven donepezil clinical trials in AD [28-30,33-36], including data from open label extensions of two of the studies [37,38]. Trial data included 2,700 patients from the US, Canada, UK, France, and five Nordic countries, with up to 52 weeks of follow-up. The inclusion of trials in the current analyses was based on several criteria. Most importantly, to develop equations related to disease progression and treatment effects, access to patient level data was required. In selecting trials to be included in the patient level analyses, studies had to be Phase III or later, had to include a measure of baseline MMSE, and had to include at least one of the effectiveness outcomes included in the model. Studies conducted in special populations (e.g., women only, Apo-E subtypes, nursing home residents only); or of open label design only were excluded, as were dose finding studies.

While MMSE data over time were available from trial data the patterns of change observed in CERAD were more consistent with previous findings on progression of AD in untreated patients [39-41] and were therefore used to model the natural history of cognitive changes in the absence of treatment. A piecewise linear regression model was fitted to the annual rate of change in MMSE. This approach allows for a different slope in different intervals of the MMSE scale to reflect differences in the rate of change at different disease stages. Variables considered included patient age at baseline, sex, disease duration, baseline MMSE, and rate of decline in the first year (labeled PrevRate). The following MMSE equation was derived, retaining variables significant at the 0.05 level:

$$\begin{array}{c} RateofChange=-5.4663-0.4299PM_{1}-0.0042PM_{2}+0.1415PM_{3}-0.0791PrevRate\\ +0.0747Age+\delta_{i} \end{array}$$

PM represents patients' previous MMSE measurement, partitioned over the MMSE scale. PM1--PM3 are calculated as: PM1 = min(PrevMMSE, 9), PM2 = max[0, min(PrevMMSE-9, 9)], and PM3 = max[0, min(PrevMMSE-18, 12)]. δi represents a random intercept parameter, allowing the pattern of decline to vary between patients. The MMSE scale itself ranges from 0 to 30.

To apply a treatment effect for donepezil, a similar model was fitted to the donepezil trial data to identify differences in rate of cognitive decline [19]. In the first 20 weeks of treatment, the estimated coefficients for treatment effect on annual rate of change was 6.16 and 2.47 over weeks 20 to 52. After 1 year, further treatment was assumed to simply maintain previous gains (i.e. the treatment term in the rate of change equation is set to 0). Note that these coefficients are not the sole determinant of treatment effect size given that rate of change in MMSE is also influenced by individuals' previous rate of decline and overall disease severity. Furthermore, the coefficients for treatment effect influence the annual rate of change, and are applied differentially depending on time on treatment and how long patients remain on treatment. In order to test the validity of the effect size calculations, simulated effect sizes at 6 months were compared to the observed effect sizes in the clinical trials [20], with the simulation resulting in an estimate of improvement of 1.92 points on the MMSE for donepezil versus no treatment, compared to the observed 1.88 point difference.

NPI was predicted based on the donepezil trials where NPI data were collected. It was modeled as change from NPI at baseline.

$$\begin{array}{c} Change_{NPI}=(5.74-0.64Donepezil+0.03Weeks-0.59NPI_{base}-0.001NPI∙Weeks\\ +0.24NPI_{recent}-1.74White-3.82Black+2.34PsyMed+0.12MMSE_{base}-0.22MMSE_{recent}+\delta_{i})∙1.44 \end{array}$$

*Donepezil *represents the treatment effect of donepezil, *Weeks *stands for weeks of follow-up in the simulation, *NPI_base _*is the patient's baseline NPI, *NPI_recent _*is the patient's last NPI. *White *and *Black *are dummy variables for race (*All Other Races *was the reference), *PsyMed *is a dummy variable for patients treated with psychiatric medications at baseline, *MMSE_base _*represents the patient's MMSE at baseline, and *MMSE_recent _*represents the patient's previous MMSE. *δ_i _*represents a random intercept parameter, which allows the pattern of decline to vary between patients. Patient age and sex, as well as rate of MMSE decline were also tested as predictors, but failed to reach a significance level of 0.05. The equation for NPI was derived based on a normalized scale of 0 to 100, and is therefore multiplied by 1.44 to rescale it to the standard 0 to 144 range for the NPI.

As changes in NPI are influenced by patients' baseline and most current MMSE, the treatment effect of donepezil is realized both through the treatment coefficient and its influence on MMSE over time [19]. The treatment effect coefficient, therefore, only partially accounts for the impact of treatment on NPI changes, as patients on treatment will generally have better MMSE scores. For example, a patient who has a 1 point treatment effect on MMSE, will experience a total treatment effect on NPI of 1.44*[(-0.22 × 1) -0.64], or -1.27.

For the scales that measure function (ADL and IADL) standardized scales ranging from 0 (best function) to 100 (worst function) were created based on the available clinical trial data.

As with NPI, ADL and IADL equations predict change from baseline:

$$\begin{array}{c} RateofChange_{ADL}=1.35-0.81Donepezil+0.06Weeks-0.79ADL_{base}+0.71ADL_{previous}\\ +0.12MMSE_{base}+0.09Age+0.81PsyMed-3.05Black-0.49MMSE_{recent}+\delta_{i} \end{array}$$

$$\begin{array}{c} RateofChange_{IADL}=1.27+0.63Donepezil+0.17Weeks-0.06Donepezil∙Weeks-0.84IADL_{base}\\ +0.002IADLWeek+0.84IADL_{previous}-0.67Male+0.20MMSE_{base}-0.28MMSE_{recent}\\ -0.16ADL_{base}+0.18ADL_{recent}+\delta_{i} \end{array}$$

*Age *stands for the patient's age at baseline in years. Potential predictors considered for inclusion (at 0.05 level) were treatment, time, baseline and most recent ADL/IADL, baseline and most recent MMSE, baseline and most recent NPI, age, sex, treatment, and use of anti-psychotic medications.

For ADL scores, donepezil's effect was modeled directly through the treatment effect and the terms for patients' most recent MMSE. For IADLs, treatment comes into play through the treatment term, as well as patients' most recent MMSE and ADL scores.

Additional technical details on the equations used in the simulation have been published elsewhere [19]. Standard errors and a validation that simulated results on treatment effect from the predictive equations and compare well to observed results on treatment effect are also available [20], although IADL treatment effect sizes were underestimated at 6 months. For NPI, the simulated treatment effect size was 1.75 points compared to the observed 1.68 for ADL, the simulated effect size was 2.55 points compared to an observed 2.59 and for IADLs, the simulated effect size was 1.69 points compared to an observed 3.79.

As head-to-head data were not available for memantine, the simulation predicts disease progression and treatment persistence for patients on memantine using the parameters for patients on donepezil, but modifies these parameters using the difference between 6-month placebo adjusted clinical trial results for memantine 20 mg and donepezil 10 mg in moderate to severe AD patients (Table 1). Data for memantine were extracted from a Cochrane meta-analysis [42].

**Table 1:** Changes in placebo-adjusted effectiveness outcomes for donepezil and memantine

Outcome	Donepezil	Memantine	Difference
MMSE (0-30)	1.16	0.48	-0.68
NPI (0-144)	-2.40	-2.76	-0.36
Function (0-100)	-4.44	-2.35	2.09
Relative Risk of Drop-Out	1.18	0.66	0.62

#### Persistence

Patients can stop treatment in the simulation either by reaching the end of the user-specified treatment duration (10 years in base case), based on clinical stopping rules (e.g., MMSE falling below 10 as explored in the sensitivity analyses), or other non-specified reasons. The analyses are based on the assumption that patients who stop treatment lose all treatment benefits over the course of the subsequent 6 weeks [37].

Hazard ratios for premature treatment discontinuation are derived from the donepezil clinical trial data and applied to base discontinuation rates derived from actual practice data in the UK [43] (Table 2) as there are no equivalent data available for Germany. The hazard ratios are based on a Cox regression model in which MMSE and the rate of decline in MMSE were updated over time. Demographic variables were also tested as predictors of discontinuation but were not significant (0.05) and not retained.

**Table 2:** Baseline discontinuation rates for patients on donepezil and memantine

	Months 0-3	Months 0-6	Months 6-12	Annual Risk After 12 Months
Donepezil	5.1%	5.1%	10.2%	10.3%
Memantine	3.1%	3.1%	6.3%	6.3%

#### Mortality

German-specific survival data were obtained from the German Federal Statistical Office [44]. As there are no disease specific survival data for Germany available, gender-specific differences in survival for the UK and German populations aged 65 years and older were applied to survival times from the Medical Research Council's cognitive function and ageing study (MRC CFAS), and time to death functions derived based on patient age and gender at baseline [45]. Mortality was assumed to be unaffected by treatment.

#### Medical costs

Daily treatment costs of €4.20 for donepezil 10 mg and €3.83 for memantine 20 mg were derived from the Rote Liste^®.^[46]. Patients on active therapy were assumed to incur costs associated with biannual visits to their physician. Costs for outpatient service provision, such as GP or specialist visits, dementia tests, laboratory tests or imaging are based on the German tariff EBM 2008 [46].

Direct patient care costs per disease state are based on a 2007 publication on the cost effectiveness of donepezil in Germany [18,47], and inflated to 2008 Euros, using the harmonized index of consumer prices for Germany [48]. It provides monthly costs by severity level which were interpolated to provide estimates for intermediate disease severity stages (Table 3). The same cost was applied regardless of location of care because health state costs included costs for both ambulatory care and nursing homes.

**Table 3:** Cost inputs

Care costs by disease severity (MMSE)		Monthly costs	Source
Mild	≥ 25	€184	Adapted from Teipel et al. 2007[18]
	≥ 20 - < 25	€593	
Moderate	≥ 15 - < 20	€1,275	
	≥ 10 - < 15	€1,958	
Severe	< 10	€2,981	

**Drugs**		**Treatment costs** per day**	**Source**

Donepezil 10 mg		€4.20	Rote Liste^® ^2008[49]
Memantine 20 mg		€3.83	Rote Liste^® ^2008[49]

**Outpatient treatment monitoring**		**Costs per quarter**	**Source**

GP visit, geriatric assessment, MMSE test (one per quarter)		€53.47*	EBM2008[46]

* Including once-quarterly lump-sum for GP visit (€37.74), geriatric assessment (€ 13.69) and MMSE test (€2.04)

** Treatment costs per day are calculated based on the N3 package price provided in the Rote Liste^® ^converted to a daily cost based on the number of tablets per pack

#### Caregiver time costs

Caregiver time was linked to disease severity parameters [20] based on an equation derived from two of the donepezil clinical trials where these data were available, and took the form of [29,30]:

$$\begin{array}{c} CareMinutesPerDay=76.41+1.8Age_{cg}^{}+93.02Male_{cg}+85.56Male_{patient}-6.47MMSE\\ +0.58NPI+2.66ADL+2.61IADL+20.55PsyMed \end{array}$$

*Age_cg _*stands for the caregiver's age, *Male_cg _*is a dummy variable for the caregiver's sex, and *Male_patient _*for the patient's sex. Patient age and relation of patient to caregiver (spouse, child, or other) were other parameters tested but dropped for lack of significance with *p *> 0.10. The *p*-value for *PsyMed *was 0.25 but it was retained as it was a confounding variable (i.e., dropping it biased the values of the other coefficients).

In sensitivity analyses, an alternative assignment of caregiver time was used, with caregiver time calculated based solely on patient's MMSE score using estimates reported for Germany [47]. Caregiver time was valued at €5.21 per hour based on a published study [18,47].

#### Location of care

Costs and time by location of care are accumulated based on the severity of disease patients experience over the course of the simulation. Similarly, time spent by patients in institutions is allotted as percentage of the time that the patient was alive. Institutionalization rate was calculated based on institutionalization rates of AD patients in Germany [50] which were reported as 42.9% for patients with MMSE scores below 20, and 0% for those with scores of 20 or higher. In order to produce a finer gradient, these results were fit to a simple linear regression which predicted the proportion of patients institutionalized as 64.35% - 2.86% × MMSE, with rates varying from 0% for those at the mildest stages of the disease to 50% for those with severe AD.

#### Health utilities

Patients' health utilities were estimated based on a previously published regression equation [51] which used the EQ-5D to derive health utilities for 272 AD patients in Nordic countries [19]. The NPI term in the published equation was based on the brief NPI, and was modified to reflect the full NPI range (0 to 144) used in the simulation. The final equation took the following form and is applied in the model by using patients' values (e.g., MMSE score) over the course of the simulation to calculate the appropriate QALYs to be assigned to that patient:

Utility=0.408+0.010MMSE-0.004NPI-0.159Institutionalized+0.051Caregiver

*MMSE *represents the patient's current MMSE, *NPI *represents the patient's current NPI. *Institutionalized *is a dummy variable for whether the patient is institutionalized. *Caregiver *indicates whether the patient lives with their caregiver.

Caregiver utilities are assigned based on equations derived from the donepezil trial data where caregivers completed the SF-36 [20,28-30]. Scores were transformed to health utilities [52] and a linear repeated measures model was used to develop the following equation:

$$\begin{array}{c} CaregiverUtility=0.90-0.003Age_{CG}+0.03Male_{CG}+0.001Age_{patient}+0.00MMSE-0.001NPI\\ -0.001ADL-0.0004IADL-0.01PsyMed \end{array}$$

Patient sex and relation of patient to caregiver (spouse, child, or other) were other variables tested but dropped for lack of significance with *p *> 0.10. *PsyMed *and *IADL *had *p*-values of 0.20 but were retained as they were confounders.

### Analyses

Base case analyses were run for patients with mild to moderate AD (26 ≥ MMSE ≥ 10) treated with donepezil 10 mg versus no treatment, and for patients with moderate AD (MMSE 10-19) on donepezil versus memantine 20 mg over a 10 year time horizon.

The following parameters were varied in the probabilistic sensitivity analyses: treatment effects on MMSE, NPI, ADL, and IADL, patient care costs, caregiver time regression parameters, patient and caregiver utility regression parameters, the proportion of patients living in the community by disease severity, and treatment discontinuation rates.

Standard errors were available for many parameters from the parameter source data, reflecting the study sampling error. Where standard errors were not available, 25% of the parameter mean was used to assign an assumed 95% confidence interval from which standard error estimates were derived. A normal distribution was assumed for parameters on continuous variables, while proportion parameters on discrete variables were assumed to be beta distributed.

## Results

In patients with mild to moderately severe AD (26 ≥ MMSE ≥ 10), donepezil dominates no treatment from both the GKV/SPV perspective, with savings averaging €7,007 per patient (€7,323 undiscounted). From the societal perspective where savings increase to €9,893 per patient (€10,384 undiscounted) (Table 4), donepezil treatment is associated with an increase in QALYs averaging 0.13 per patient (0.14 undiscounted). For caregivers, donepezil treatment increases QALYs by 0.01 compared to caregivers of untreated patients (0.02 undiscounted). Donepezil also increases the amount of time patients spend with MMSE scores above 10 by an average of 24 weeks per patient, NPI scores below 28 by almost 6 weeks, and ADL/IADL scores below 50 by more than 7 and 3 weeks, respectively. In patients with moderate including moderately-severe AD (20 > MMSE ≥ 10), donepezil also dominates memantine, although savings are smaller, averaging €1,960 per patient (€2,097 undiscounted) from the GKV/SPV- and €2,825 per patient (€3,012 undiscounted) from the societal perspective. QALY gains are clearly smaller, with donepezil associated with an average QALY gain of 0.01 per patient versus memantine (0.01 undiscounted), and caregiver QALYs gained at less than < 0.001. The reduction in time patients spend institutionalized also falls by just over 10 days per patient. For patients with moderately severe AD, donepezil still dominates no treatment, although consistent with findings in the UK [20]. Cross reference Getsios 2010, per patients savings were lower at €8,043, as were both patient and caregiver QALYs gained, at 0.120 and 0.013, respectively. Memantine also led to lower overall costs and improved QALYs relative to no treatment, although both savings and QALYs gained, although savings were 35% lower compared to those with donepezil, and QALYs gained 13% lower.

**Table 4:** Base case results by disease severity for the 10 years following treatment initiation*

Patients with MMSE ≥ 10 and ≤ 26 versus untreated patients	Untreated	Donepezil	Net difference
Survival (undiscounted, in years)	4,870	4,870	0,000
Drug Costs	€ 0	€ 4,625	€4,625
Total Non-Drug Direct Costs	€126,863	€115,231	-€11,632
Total Direct Costs	€126,863	€119,856	-€7,007
Indirect Costs	€87,138	€84,253	-€2,885
**Total Costs**	**€214,001**	**€204,108**	**-€9,893**
Years with MMSE > 10	1,972	2,435	0,463
Years with NPI < 28	2,680	2,794	0,114
Years with ADL < 50	1,896	2,036	0,140
Years with IADL < 50	0,241	0,303	0,062
Years in Institution	1,663	1,457	-0,206
Total Care Time (Years)	1,908	1,845	-0,063
QALYs (Patient)	1,659	1,790	0,131
QALYs (Caregiver)	3,272	3,287	0,014
**QALYS (Patient + Caregiver)**	**4,931**	**5,077**	**0,146**
Health Care Direct Cost/QALY (Patient + Caregiver)		Dominant	
Societal Total Cost/QALY (Patient +Caregiver)		Dominant	

**Patients with MMSE ≥ 10 and < 20 versus memantine**	**Memantine**	**Donepezil**	**Net difference**

Survival (undiscounted, in years)	4,909	4,909	0,000
Drug Costs	€4,972	€4,696	-€276
Total Non-Drug Direct Costs	€129,702	€128,019	-€1,684
Total Direct Costs	€134,674	€132,715	-€1,960
Indirect Costs	€89,572	€88,707	-€ 865
**Total Costs**	**€224,246**	**€221,422**	**-€2,825**
Years with MMSE > 10	2,023	2,801	0,058
Years with NPI < 28	2,808	2,751	-0,058
Years with ADL < 50	1,772	1,835	0,062
Years with IADL < 50	0,186	0,209	0,023
Years in Institution	1,702	1,673	-0,028
Total Care Time (Years)	1,961	1,942	-0,019
QALYs (Patient)	1,663	1,677	0,014
QALYs (Caregiver)	3,276	3,279	0,003
**QALYS (Patient + Caregiver)**	**4,939**	**4,956**	**0,017**
Health Care Direct Cost/QALY (Patient + Caregiver)		Dominant	
Societal Total Cost/QALY (Patient +Caregiver)		Dominant	

*All outcomes are presented at discounted values

In one-way sensitivity analyses key parameters such as caregiver time, costs, utilities, institutionalization and treatment effects were varied. Regardless of the variation, donepezil remained dominant compared to both no therapy and memantine. Rates of treatment discontinuation and the duration of treatment had the strongest influence on the extent of savings and health benefits (Table 5).

**Table 5:** One-way sensitivity analyses

Analysis	QALYs	Total costs	Cost per QALY	QALYs	Total costs	Cost per QALY
	Patients with MMSE 10-26: donepezil versus no treatment			Patients with MMSE 10-20: donepezil versus memantine		
**Base Case**	**0.146**	**-€9,893**	**Dominant**	**0.017**	**-€2,825**	**Dominant**
Caregiver Time Effects of Disease Severity ↓ 25%^†^	0.146	-€9,171	Dominant	0.017	-€2,608	Dominant
Patient Care Cost ↓ 25%^‡^	0.146	-€6,911	Dominant	0.017	-€2,417	Dominant
Patients institutionalized ↓ 25%	0.135	-€9,893	Dominant	0.015	-€2,825	Dominant
Patient Utility Effects of Disease Severity ↓ 25%	0.124	-€9,893	Dominant	0.015	-€2,825	Dominant
Stop Treatment if MMSE < 10	0.132	-€11,006	Dominant	0.018	-€2,279	Dominant
Stop Treatment if MMSE Deteriorates on any Scale after 6 Months	0.140	-€9,691	Dominant	0.054	-€4,578	Dominant
5 Year Time Horizon	0.140	-€10,172	Dominant	0,016	-€2,578	Dominant
Treatment Effects ↓ 25%^†^	0.107	-€5,930	Dominant	0.023	-€3,379	Dominant
No Discontinuation	0.204	-€13,215	Dominant	0.041	-€4,301	Dominant
Double Discontinuation	0.106	-€7,412	Dominant	0.003	-€1,760	Dominant
Treatment Duration 5 Years	0.140	-€10,171	Dominant	0.016	-€2,580	Dominant
Treatment Duration 1 Year	0.042	-€2,704	Dominant	0.008	-€991	Dominant
Alternative caregiver time effect (German data)[47]	0.146	-€16,677	Dominant	0.017	-€3,288	Dominant
Alternate disease severity definition based on German convention MMSE ranges *	0.151	-€9,486	Dominant	0.019	-€3,448	Dominant

^† ^Coefficients in regression equations relating to disease severity (MMSE, NPI, ADL, and IADL) were reduced by 25%

^‡ ^All patient care costs were reduced by 25%

* Mild MMSE ≥ 18, moderate MMSE 10-17, severe MMSE < 10

**Figure 2:**
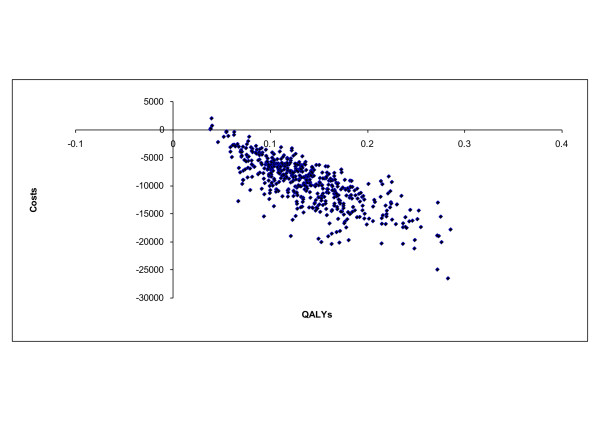
**Cost-effectiveness scatter plot for patients with 26 ≥ MMSE ≥ 10: donepezil versus no treatment**. Distribution of replications: 0.8% in upper right quadrant (donepezil leads to incremental costs and higher QALYs), 99.2% in lower right quadrant (donepezil dominant, leading to lower costs and higher QALYs).

**Figure 3:**
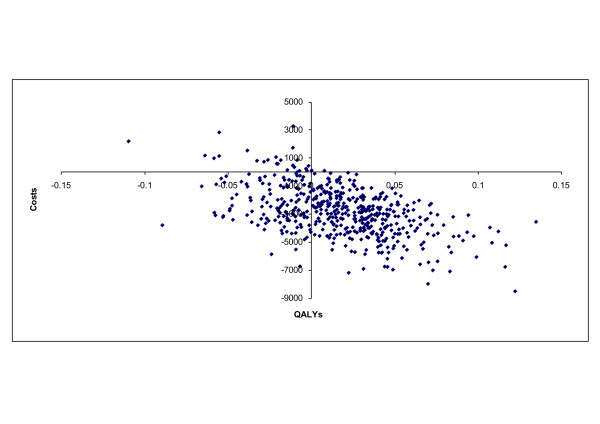
**Cost-effectiveness scatter plot for patients with 20 > MMSE ≥ 10: donepezil versus memantine**. Distribution of replications: 69.8% in lower right quadrant (donepezil dominant, leading to lower costs and higher QALYs), 25.2% in lower left quadrant (donepezil leads to lower costs and lower QALYs), 4.6% in upper left quadrant (donepezil dominated, leading to higher costs and lower QALYs), and 0.4% in the upper right quadrant (donepezil leads to higher costs and higher QALYs).

In probabilistic sensitivity analyses, donepezil dominates no treatment in almost all replications from both the health care payer and societal perspectives (Figure 2). Versus memantine, donepezil dominates in 70% of replications, and leads to savings in 95% of replications (Figure 3). For the analyses versus memantine, at a threshold of 10,000 Euro/QALY, donepezil was cost-effective from both perspectives in over 90% of replications.

## Discussion

The discrete event simulation developed for donepezil provides a flexible framework for the assessment of treating AD patients with donepezil. By integrating patients' individual characteristics, heterogeneity in the population, disease progression and outcomes can be captured. Disease progression was modeled not solely relying on 1 year RCT data, but also using longer term CERAD registry data, which allows for a more realistic representation of the disease course. The model allows for analysis of population subgroups, with different settings for time horizon, treatment duration, discontinuation rules, and treatment effects. Cost, utility and caregiver inputs can be specified for different severity ranges and locations of care, or can be specified using predictive equations, making adaptation of the model and incorporation of new data easier.

The analyses for Germany indicate that donepezil is clearly cost-effective in the treatment of patients with mild to moderately-severe AD. In the base case and all one-way sensitivity analyses, donepezil dominated no treatment and memantine in all scenarios evaluated. Results of the probabilistic sensitivity analyses were also highly favorable with donepezil dominating no treatment in virtually all replications, and leading to savings in the comparison with memantine in 95% of replications. With the availability of generic cholinesterase inhibitors, cost savings should be even greater, although the contribution of the cost of treatment with donepezil to overall costs is modest, representing less than 2.5% of total costs in patients with mild to moderately-severe AD.

Our model differs from most previous economic models in AD in that we model disease progression over several domains using continuous scales, rather than using a single domain and/or limiting outcomes to a small number of discrete health states. Although more favorable overall with donepezil predicted to dominate no treatment, the results of this study are much more in line with previous findings for Germany [19], where a base-case ICER of €4,264 per QALY for donepezil versus placebo was estimated. That study also indicated that starting treatment early leads to cost reductions and therefore improved cost-effectiveness. The more favorable predictions from our simulation are chiefly a result of the greater sensitivity of our model in capturing changes in cognition, and in the case of indirect costs, the consideration of not only cognition, but also patient function and behavioral symptoms.

The current simulation is not without limitations. For example, the longest-duration of head to head clinical trial data available was for 1 year versus placebo [29]. With the assumption that continued treatment after 1 year serves as a maintenance function only with no further slowing of the rate of disease progression, we have adopted a conservative approach consistent with most other modeling studies in this area. Furthermore, we assume that all benefits are lost within 6 weeks if treatment is discontinued. Comparisons with memantine are subject to even greater uncertainty, as no head-to-head trial data are available, requiring indirect comparisons based on pooled trial results. Clearly, incorporation of head to head clinical trial data versus memantine would strengthen comparisons and yield more robust results. In addition, the current analyses evaluate memantine monotherapy versus donepezil monotherapy. Although memantine also be used as an add-on treatment to cholinesterase inhibitors, this was not evaluated, as the focus of these analyses was on the cost-effectiveness of donepezil, and not memantine.

The source population for the trial data was not German, though it was weighted for the age and sex distribution of German AD patients when defining the simulated population. Other limitations of the data revolve around assigning costs and utilities associated with different degrees of disease severity. A recent review paper on health utilities [53] used in economic evaluations, noted the limited amount of data on severity specific health utilities in populations with AD, and the poor correlation between patient-based utilities, and those derived by caregiver proxy.

The cost data for Germany are based entirely on MMSE ranges (i.e., they do not consider behavior or function). Furthermore published German cost data for AD at the time of the analysis were scant, with only one publication providing suitable information [47]. A number of studies on dementia and AD costs were published in 2011 [52,54-56]. Those studies that did report costs by severity of disease [52,54,55], all found that costs increase markedly with increased disease severity, consistent with the data used in the current model and therefore would not alter our conclusions. The one study that examined costs in patients with AD, for example [54] found that the annual costs of the disease average €13,080 per patient per year (€ 2009), but approached €25,000 per patient for those at the most severe stages of the disease, compared to well under €7,000 per patient for those at the most mild stages. Finally, although wide variation exists in the valuation of informal care, with donepezil dominant over no treatment and memantine, even when these costs are considered, the method of assigning costs to caregiver time would only influence the extent of savings associated with donepezil. Of note, however, the hourly costs assigned to caregiver time are substantially lower than those used in recent German costing studies in dementia [52,54,55].

## Conclusions

These analyses indicate that donepezil is highly cost-effective in the treatment of mild to moderate AD in Germany, and is likely associated with significant cost savings when compared to untreated patients. While benefits over memantine are modest, the base case and sensitivity analyses results suggest a high likelihood that donepezil would lead to cost savings if used in place of memantine.

## Abbreviations

CDR: Clinical Dementia Rating Scale; MMSE: Mini-Mental State Examination; AD: Alzheimer's disease; NPI: Neuropsychiatric Inventory; ADLs: Activities of daily living; IADL: Instrumental activities of daily living; CERAD: Consortium to Establish a Registry for Alzheimer's Disease; MRC CFAS: Medical Research Council's cognitive function and ageing study.

## Competing interests

DG, ST, SB (SH is a former employee) are all employees of a consulting company, which has conducted research on behalf of Eisai, including research on donepezil. They have not received any reimbursements, fees, funding, or salary from an organization that could gain or lose financially from the publication of this manuscript. Partial funding for this manuscript was provided by Eisai, and Eisai will be financing the processing charge. Eisai reviewed the manuscript and Grant Maclaine, one of the co-authors was an employee of Eisai at the time of manuscript preparation. Eisai placed no restrictions on the content of the manuscript. They do not hold any stocks or shares. The only data used in the study that are not publicly available are those from the donepezil clinical trials (although results from these trials are all publicly available). Ethics approval for these trials had been obtained.

GM was an employee of Eisai Europe Limited at the time of preparation of the manuscript. Eisai provided funding for manuscript development and will pay the manuscript processing charge. Data, other than those from the donepezil clinical trials, are publicly available. Results from the donepezil clinical studies have been separately published and ethics committee approvals were received for these studies.

## Authors' contributions

SH participated in the drafting of the manuscript, data collection, contribution to model analyses and interpretation of results. DG participated in the Model concept/design, data analyses and interpretation, critical revision of article. SB contributed to model design and implementation, data analyses, and drafting of the manuscript. ST contributed to the data/model analyses and critical revision of article. GM contributed to the model concept/design, critical revision/approval of the article (funding approved in cooperation with German Eisai affiliate).

## Funding

This research was funded by Eisai GmbH, Germany. Eisai GmbH was informed throughout the process of data collection and analyses, and contributed to the research through critical review of results and manuscript.

## Pre-publication history

The pre-publication history for this paper can be accessed here:

http://www.biomedcentral.com/1471-2377/12/2/prepub
